# Supplementary material for: Exploring the barriers and facilitators to the uptake of smoking cessation services for people in treatment or recovery from problematic drug or alcohol use: A qualitative systematic review
Source: PLoS One. 2023 Jul 13;18(7):e0288409. doi: 10.1371/journal.pone.0288409 (PMC10343091; doi:10.1371/journal.pone.0288409)
Supplement: S1 Appendix — (PDF) [file pone.0288409.s001.pdf]

NHS
National Institute for Health Research

UNIVERSITY of York
Centre for Reviews and Dissemination

Systematic review

A list of fields that can be edited in an update can be found here

1. * Review title. [1 change]

Give the title of the review in English

Exploring the barriers and facilitators to the uptake of smoking cessation services for people in treatment or recovery from problematic drug or alcohol use: a qualitative systematic review

2. Original language title.

For reviews in languages other than English, give the title in the original language. This will be displayed with the English language title.

3. * Anticipated or actual start date.

Give the date the systematic review started or is expected to start.

15/11/2021

4. * Anticipated completion date. [2 changes]

Give the date by which the review is expected to be completed.

31/01/2023

5. * Stage of review at time of this submission.

This field uses answers to initial screening questions. It cannot be edited until after registration.

Tick the boxes to show which review tasks have been started and which have been completed.

Update this field each time any amendments are made to a published record.

The review has not yet started: No

**Review stage**	**Started**	**Completed**
Preliminary searches	Yes	Yes
Piloting of the study selection process	Yes	No
Formal screening of search results against eligibility criteria	Yes	No
Data extraction	No	No
Risk of bias (quality) assessment	No	No
Data analysis	No	No

Provide any other relevant information about the stage of the review here.

6. * Named contact.

The named contact is the guarantor for the accuracy of the information in the register record. This may be any member of the review team.

Eric Osayemwenre Iyahen

Email salutation (e.g. "Dr Smith" or "Joanne") for correspondence:

Mr Iyahen

7. * Named contact email.

Give the electronic email address of the named contact.

ericiyahen@gmail.com

8. Named contact address [1 change]

Give the full institutional/organisational postal address for the named contact.

Ministry of Health, Block D, Secretariat Building, PMB 1123, Sapele Road, Benin City, Edo State, Nigeria

9. Named contact phone number.

Give the telephone number for the named contact, including international dialling code.

+2348063448954

10. * Organisational affiliation of the review.

Full title of the organisational affiliations for this review and website address if available. This field may be completed as 'None' if the review is not affiliated to any organisation.

The University of Edinburgh

Organisation web address:

https://www.ed.ac.uk

11. * Review team members and their organisational affiliations.

Give the personal details and the organisational affiliations of each member of the review team. Affiliation refers to groups or organisations to which review team members belong. **NOTE: email and country now MUST be entered for each person, unless you are amending a published record.**

Mr Eric Iyahen. Ministry of Health, Edo State, Nigeria
Dr Fiona Dobbie. The University of Edinburgh
Mr Osazee Omoruyi. Ministry of Health, Edo State, Nigeria

12. * Funding sources/sponsors.

Details of the individuals, organizations, groups, companies or other legal entities who have funded or sponsored the review.

None

Grant number(s)

State the funder, grant or award number and the date of award

13. * Conflicts of interest.

List actual or perceived conflicts of interest (financial or academic).

None

14. Collaborators.

Give the name and affiliation of any individuals or organisations who are working on the review but who are not listed as review team members. **NOTE: email and country must be completed for each person, unless you are amending a published record.**

Dr Neneh Rowa-Dewar. University of Edinburgh

15. * Review question. [1 change]

State the review question(s) clearly and precisely. It may be appropriate to break very broad questions down into a series of related more specific questions. Questions may be framed or refined using PI(E)COS or similar where relevant.

1. What barriers do people with problematic drug or alcohol use face when seeking help to cut down or stop smoking?

2. What factors promote the successful engagement and positive experiences of interaction with stop

smoking services for people with/recovering from problematic drug or alcohol use?

16. * Searches. [1 change]

State the sources that will be searched (e.g. Medline). Give the search dates, and any restrictions (e.g. language or publication date). Do NOT enter the full search strategy (it may be provided as a link or attachment below.)

The following searches will be undertaken:

1. PubMed - 19th November 2022;

2. Embase (Ovid) - 19th November 2022;

3. PsycINFO (Ovid) - 19th November 2022;

4. Cumulative Index to Nursing and Allied Health Literature Plus [CINAHL Plus] (EBSCOhost) - 19th November 2022.

Restrictions:

Studies published in English

Studies having humans as subjects

No restriction by publication date or type, as qualitative papers are poorly indexed in most databases

17. URL to search strategy.

Upload a file with your search strategy, or an example of a search strategy for a specific database, (including the keywords) in pdf or word format. In doing so you are consenting to the file being made publicly accessible. Or provide a URL or link to the strategy. Do NOT provide links to your search **results**.

Alternatively, upload your search strategy to CRD in pdf format. Please note that by doing so you are consenting to the file being made publicly accessible.

Do not make this file publicly available until the review is complete

18. * Condition or domain being studied. [1 change]

Give a short description of the disease, condition or healthcare domain being studied in your systematic review.

Smoking cessation intervention for people with/recovering from problematic drug or alcohol use

19. * Participants/population.

Specify the participants or populations being studied in the review. The preferred format includes details of both inclusion and exclusion criteria.

The participants are people who are in treatment or recovery from problematic drug or alcohol use and those involved in providing treatments.

20. * Intervention(s), exposure(s). [1 change]

Give full and clear descriptions or definitions of the interventions or the exposures to be reviewed. The preferred format includes details of both inclusion and exclusion criteria.

The intervention will be any type of tobacco smoking cessation support or services delivered at any kind of free stop smoking service during problematic drug or alcohol use treatment/recovery

21. * Comparator(s)/control.

Where relevant, give details of the alternatives against which the intervention/exposure will be compared (e.g. another intervention or a non-exposed control group). The preferred format includes details of both inclusion and exclusion criteria.

Not applicable.

22. * Types of study to be included. [1 change]

Give details of the study designs (e.g. RCT) that are eligible for inclusion in the review. The preferred format includes both inclusion and exclusion criteria. If there are no restrictions on the types of study, this should be stated.

Qualitative studies, mixed method studies (qualitative component only), or RCTs with qualitative components in the process evaluation, will be included.

23. Context.

Give summary details of the setting or other relevant characteristics, which help define the inclusion or exclusion criteria.

24. * Main outcome(s).

Give the pre-specified main (most important) outcomes of the review, including details of how the outcome is defined and measured and when these measurement are made, if these are part of the review inclusion criteria.

Establishing the barriers and facilitators to uptake of smoking cessation support for people with/recovering from drug or alcohol problem, to successfully cut down or reduce smoking.

Measures of effect

Please specify the effect measure(s) for you main outcome(s) e.g. relative risks, odds ratios, risk difference, and/or 'number needed to treat.

25. * Additional outcome(s).

List the pre-specified additional outcomes of the review, with a similar level of detail to that required for main outcomes. Where there are no additional outcomes please state 'None' or 'Not applicable' as appropriate to the review

None.

Measures of effect

Please specify the effect measure(s) for you additional outcome(s) e.g. relative risks, odds ratios, risk difference, and/or 'number needed to treat.

[1. change]

26. * Data extraction (selection and coding).

Describe how studies will be selected for inclusion. State what data will be extracted or obtained. State how this will be done and recorded.

Upon successful completion of piloted and finalized search terms; the references will be exported to Covidence which is an online systematic review software program which facilitates collaborative work by multiple reviewers through the process of screening, full-text review, data extraction and risk of bias assessment. Duplicated records will be automatically identified and removed by Covidence, after which the principal reviewer (Eric Iyahen) will review the titles of the remaining articles to remove topics that are unrelated to the review objectives. 10% of the articles deemed 'irrelevant' will be checked="checked" value="1" by a second reviewer (Osaze Otemuyiwa) to ensure objectivity.

Titles and abstracts of articles that passed the 'title only' check, will be independently screened by both principal and second reviewer based on a pre-defined screening criteria documented in Covidence, Studies that potentially meet the eligibility criteria will undergo full-text screening by both the principal and second reviewer. Reasons for exclusion will be documented in Covidence for inclusion in the PRISMA flow diagram of the systematic review processes.

A data-extraction template will be created using Microsoft Word and tested with three of the included study articles, by both reviewers. The template will be used to record the aims of research, country where research was conducted, the participants' description and demography, treatment or recovery status of participants, qualitative research framework and key findings. These information will be stored using Microsoft Word document for ease of sharing via email rather than suing a reference manager.

In all cases, where the principal and second reviewer fails to reach a consensus; another member of the review team (Fiona Dobbie) will be called upon to mediate and bring resolution. The third independent reviewer is a doctoral research fellow in the field of public health at the University of Edinburgh.

27. * Risk of bias (quality) assessment.

State which characteristics of the studies will be assessed and/or any formal risk of bias/quality assessment tools that will be used.

The critical appraisal checklist developed by the National Institute for Health and Care Excellence (NICE) for qualitative studies will be used to review the selected articles. For each qualitative study, the tool will assess 14 items under the key domains such as the theoretical approach and clarity of its aims; the rigour of the methods; how well the data collection was done; the richness of the data and rigour and reliability of the analysis and findings. This will be done by the principal and second reviewers in a double-blinded assessment strategy.

An overall assessment of the study's relevance and one of three final gradings ('++', '+' or '-') will be given according to how many of the checklist criteria have been fulfilled, and if not fulfilled, whether the conclusions are likely to alter or not.

28. * Strategy for data synthesis. [1 change]

Describe the methods you plan to use to synthesise data. This **must not be generic text** but should be **specific to your review** and describe how the proposed approach will be applied to your data. If meta-analysis is planned, describe the models to be used, methods to explore statistical heterogeneity, and software package to be used.

A thematic analysis of study findings will be done. This will follow an inductive approach to make sure findings are as close to the data as possible. Published qualitative data from the included studies will be coded by the principal reviewer, with the second reviewer undertaking independent coding of three of the included studies. Lines of 'first-level' codes will be targeted towards summarizing the meaning of the text or capturing the authors' original language as much as possible. Coding will be identified as quoted original data or author interpretation. Data from service users and treatment providers will be coded and analyzed separately, indicating whether or not the data were from service users or treatment providers. Data synthesis will involve organization of first level codes into second level descriptive themes. This will be followed by summarizing first level codes whilst making sure data remain close to the included articles. Third level analytical themes will then be developed. This stage will involve capturing the line of argument of both service users and providers, beyond the first and second level codes. This will aid the generation of new findings (final themes) from the pooled data. This synthesis will follow the inductive thematic approach such that the data will reveal additional themes which are broader than the research questions because perception is relative to experiences and are not usually convergent. This will ensure that all relevant data beyond the research questions are identified.

29. * Analysis of subgroups or subsets.

State any planned investigation of 'subgroups'. Be clear and specific about which type of study or participant will be included in each group or covariate investigated. State the planned analytic approach.

None planned.

30. * Type and method of review.

Select the type of review, review method and health area from the lists below.

Type of review

Cost effectiveness

No

Diagnostic

No

Epidemiologic

Yes

Individual patient data (IPD) meta-analysis

No

Intervention

Yes

Living systematic review

No

Meta-analysis

No

Methodology

No

Narrative synthesis

No

Network meta-analysis

No

Pre-clinical

No

Prevention

No

Prognostic

No

Prospective meta-analysis (PMA)

No

Review of reviews

No

Service delivery

Yes

Synthesis of qualitative studies

No

Systematic review
Yes

Other
No

Health area of the review

Alcohol/substance misuse/abuse
Yes

Blood and immune system
No

Cancer
No

Cardiovascular
No

Care of the elderly
No

Child health
No

Complementary therapies
No

COVID-19
No

Crime and justice
No

Dental
No

Digestive system
No

Ear, nose and throat
No

Education
No

Endocrine and metabolic disorders
No

Eye disorders

No

General interest

No

Genetics

No

Health inequalities/health equity

No

Infections and infestations

No

International development

No

Mental health and behavioural conditions

No

Musculoskeletal

No

Neurological

No

Nursing

No

Obstetrics and gynaecology

No

Oral health

No

Palliative care

No

Perioperative care

No

Physiotherapy

No

Pregnancy and childbirth

No

Public health (including social determinants of health)

Yes

Rehabilitation

No

Respiratory disorders

No

Service delivery

Yes

Skin disorders

No

Social care

No

Surgery

No

Tropical Medicine

No

Urological

No

Wounds, injuries and accidents

No

Violence and abuse

No

31. Language.

Select each language individually to add it to the list below, use the bin icon to remove any added in error.

English

There is not an English language summary

32. * Country.

Select the country in which the review is being carried out. For multi-national collaborations select all the countries involved.

Nigeria

Scotland

33. Other registration details.

Name any other organisation where the systematic review title or protocol is registered (e.g. Campbell, or The Joanna Briggs Institute) together with any unique identification number assigned by them. If extracted data will be stored and made available through a repository such as the Systematic Review Data Repository (SRDR), details and a link should be included here. If none, leave blank.

34. Reference and/or URL for published protocol.

If the protocol for this review is published provide details (authors, title and journal details, preferably in Vancouver format)

Add web link to the published protocol.

Or, upload your published protocol here in pdf format. Note that the upload will be publicly accessible.

No I do not make this file publicly available until the review is complete

Please note that the information required in the PROSPERO registration form must be completed in full even if access to a protocol is given.

35. Dissemination plans.

Do you intend to publish the review on completion?

Yes

Give brief details of plans for communicating review findings.?

A paper will be submitted to a leading journal in this field.

36. Keywords. [1 change]

Give words or phrases that best describe the review. Separate keywords with a semicolon or new line. Keywords help PROSPERO users find your review (keywords do not appear in the public record but are included in searches). Be as specific and precise as possible. Avoid acronyms and abbreviations unless these are in wide use.

Barriers; Facilitators; Smoking cessation; Uptake; Drug dependence Substance abuse or misuse; Treatment or recovery

37. Details of any existing review of the same topic by the same authors.

If you are registering an update of an existing review give details of the earlier versions and include a full bibliographic reference, if available.

38. * Current review status.

Update review status when the review is completed and when it is published.New registrations must be ongoing so this field is not editable for initial submission.

Please provide anticipated publication date

Review_Ongoing

39. Any additional information. [1 change]

Provide any other information relevant to the registration of this review.

This review is being undertaken as one of the key objectives of a larger research by a consortium of researchers in the United Kingdom, to drive smoking cessation policy

40. Details of final report/publication(s) or preprints if available.

Leave empty until publication details are available OR you have a link to a preprint (NOTE: this field is not editable for initial submission). List authors, title and journal details preferably in Vancouver format.

Give the link to the published review or preprint.
