# Supplementary material for: Exploring the barriers and facilitators to the uptake of smoking cessation services for people in treatment or recovery from problematic drug or alcohol use: A qualitative systematic review
Source: PLoS One. 2023 Jul 13;18(7):e0288409. doi: 10.1371/journal.pone.0288409 (PMC10343091; doi:10.1371/journal.pone.0288409)
Supplement: S2 Appendix — (DOCX) [file pone.0288409.s002.docx]

| **Study identification: Include author, title, reference, year of**  **publication** |  |  |  |  |  |  |  |
| --- | --- | --- | --- | --- | --- | --- | --- |
| **Theoretical approach** | | | | | | | |
| 1. Is a qualitative approach appropriate?  For example:  Does the research question seek to understand processes or structures, or illuminate subjective experiences or meanings?  Could a quantitative approach better have addressed the research question?  *Appropriate*  *Inappropriate*  *Not sure* | A qualitative approach can be judged to be appropriate when the research sets out to investigate phenomena which are not easy to accurately quantify or measure, or where such measurement would be arbitrary and inexact. If clear numerical measures could reasonably have been put in place then consider whether a quantitative approach may have been more appropriate. This is because most qualitative research seeks to explain the meanings which social actors use in their everyday lives rather than the meanings which the researchers bring to the situation. Qualitative research in public health commonly measures:   - personal/lives experiences (for example, of a condition, treatment, situation) - processes (for example, action research, practitioner/patient views on the acceptability of using new technology) - personal meanings (for example, about death, birth, disability) - interactions/relationships (for example, the quality of the GP/patient relationship, the - openness of a psychotherapeutic relationship) - service evaluations (for example, what was good/bad about patients experiences of a smoking cessation group). | | | | | | |
| 2. Is the study clear in what it seeks to do?  For example:  Is the purpose of the study discussed – aims/objectives/  research question/s?  Is there adequate/appropriate reference to the literature?  Are underpinning values/assumptions/theory discussed?  *Clear*  *Unclear*  *Mixed* | Qualitative research designs tend to be theory generative rather than theory testing; therefore it is unlikely that a research question will be found in the form of a hypothesis or null hypothesis in the way that you would expect in conventional quantitative research. This does not mean however that the paper should not set out early and clearly what it is that the study is investigating and what the parameters are for that. The research question should be set in context by the provision of an adequate summary of the background literature and of the study's underpinning values and assumptions. | | | | | | |
| **Study design** | | | | | | | |
| 3. How defensible/rigorous is the research design/ methodology?  For example:  Is the design appropriate to the research question?  Is a rationale given for using a qualitative approach?  Are there clear accounts of the rationale/justification for the sampling, data collection and data analysis techniques used?  Is the selection of cases/sampling strategy theoretically justified?  *Defensible*  *Indefensible*  *Not sure* | There are a large number of qualitative methodologies, and a tendency in health to 'mix' aspects of different methodologies or to use a generic qualitative method. From a qualitative perspective, none of this compromises the quality of a study as long as:   - The research design captures appropriate data and has an appropriate plan of analysis for the subject under investigation. There should be a clear and reasonable justification for the methods chosen. - The choice of sample and sampling method should be clearly set out, (ideally including any shortcomings of the sample) and should be reasonable. It is important to remember that sampling in qualitative research can be purposive and should not be random. Qualitative research is not experimental, does not purport to be generalisable, and therefore does not require a large or random sample. People are usually 'chosen' for qualitative research based on being key informers. | | | | | | |
| **Data collection** | | | | | | | |
| 4. How well was the data collection carried out?  For example:  Are the data collection methods clearly described?  Were the appropriate data collected to address the research question?  Was the data collection and record keeping systematic?  *Appropriately*  *Inappropriately*  *Not sure/ inadequately*  *reported* | Were the method of data collection the most appropriate given the aims of the research? Was the data collection robust, are there details of:   - How the data were collected? - How the data were recorded and transcribed (if verbal data)? - How the data were stored? - What records were kept of the data collection? | | | | | | |
| **Trustworthiness** | | | | | | | |
| 5. Is the role of the researcher clearly described?  Has the relationship between the researcher and the participants been adequately considered?  Does the paper describe how the research was explained and presented to the participants?  *Clearly described*  *Unclear*  *Not described* | The researcher should have considered their role in the research either as reader, interviewer, or observer for example. This is often referred to as 'reflexivity'. It is important that we can determine: a clear audit trail from respondent all the way through to reporting, why the author reported what they did report, and that we can follow the reasoning from the data to the final analysis or theory.  The 'status' of the researcher can profoundly affect the data, for example, a middle aged woman and a young adult male are likely to get different responses to questions about sexual activity if they interview a group of teenage boys. It is important to consider age, gender, ethnicity and 'insider' status (where the interviewer/researcher is part of the group being researched or has the same condition/illness, for example). The researcher can also profoundly influence the data by use of questions, opinions and judgments, so it is important to know what the researchers' position is in that regard and how the researcher introduced and talked about the research with the participants. | | | | | | |
| 6. Is the context clearly described?  For example:  Are the characteristics of the participants and settings clearly defined?  Were observations made in a sufficient variety of  Circumstances?  Was context bias considered?  *Clear*  *Unclear*  *Not sure* | It is important when gauging the validity of qualitative data to engage with the data in a meaningful way, and to consider whether the data are plausible/realistic. To make an accurate assessment of this it is important to have information about the context of the research, not only in terms of the physical context – for example, youth club, GP surgery, gang headquarters, who else was there (discussion with parents present or discussion with peers present are likely to cause the participant to position himself very differently and thus to respond very differently) – but also in terms of feeling that the participants are described in enough detail that the reader can have some sort of insight into their life/situation. Any potential context bias should be considered. | | | | | | |
| 7. Were the methods reliable?  For example:  Was data collected by more than 1 method?  Is there justification for triangulation, or for not  triangulating?  Do the methods investigate what they claim to?  *Reliable*  *Unreliable*  *Not sure* | It is important that the method used to collect the data is appropriate for the research question, and that the data generated map well onto the aims of the study. Ideally, more than 1 method should have been used to collect data, or there should be some other kind of system of comparison which allows the data to be compared. This is referred to as triangulation. | | | | | | |
| **Analysis** | | | | | | | |
| 8. Is the data analysis sufficiently rigorous?  For example:  Is the procedure explicit – i.e. is it clear how the data was analysed to arrive at the results?  How systematic is the analysis, is the procedure reliable/dependable?  Is it clear how the themes and concepts were derived from the data?  *Rigorous*  *Not rigorous*  *Not sure/not*  *reported* | The main way to assess this is by how clearly the analysis is reported and whether the analysis is approached systematically. There should be a clear and consistent method for coding and analyzing data, and it should be clear how the coding and analytic strategies were derived. Above all, these must be reasonable in light of the evidence and the aims of the study. Transparency is the key to addressing the rigour of the analysis. | | | | | | |
| 9. Is the data 'rich'?  For example:  How well are the contexts of the data described?  Has the diversity of perspective and content been explored?  How well has the detail and depth been demonstrated?  Are responses compared and contrasted across groups/sites?  *Rich*  *Poor*  *Not sure/not*  *reported* | Qualitative researchers use the adjective 'rich' to describe data which is in-depth, convincing, compelling and detailed enough that the reader feels that they have achieved some level of insight into the research participants experience. It's also important to know the 'context' of the data, that is, where it came from, what prompted it and what it pertains to. | | | | | | |
| 10. Is the analysis reliable?  For example:  Did more than 1 researcher theme and code transcripts/  data?  If so, how were differences resolved?  Did participants feedback on the transcripts/data if possible  and relevant?  Were negative/discrepant results addressed or ignored?  *Reliable*  *Unreliable*  *Not sure/not*  *reported* | The analysis of data can be made more reliable by setting checks in place. It is good practice to have sections of data coded by another researcher, or at least have a second researcher check the coding for consistency. Participants may also be allowed to verify the transcripts of their interview (or other data collection, if appropriate). Negative/discrepant results should always be highlighted and discussed. | | | | | | |
| 11. Are the findings convincing?  For example:  Are the findings clearly presented?  Are the findings internally coherent?  Are extracts from the original data included?  Are the data appropriately referenced?  Is the reporting clear and coherent?  *Convincing*  *Not convincing*  *Not sure* | In qualitative research, the reader should find the results of the research convincing, or credible. This means that the findings should be clearly presented and logically organised, that they should not contradict themselves without explanation or consideration and that they should be clear and coherent.  Extracts from original data should be included where possible to give a fuller sense of the findings, and these data should be appropriately referenced – although you would expect data to be anonymised, it still needs to be referenced in relevant ways, for example if gender differences were important then you would expect extracts to be marked male/female. | | | | | | |
| 12. Are the findings relevant to the aims of the study?  *Relevant*  *Irrelevant*  *Partially relevant* | This section is self-explanatory. | | | | | | |
| 13. Conclusions  For example:  How clear are the links between data, interpretation and  conclusions?  Are the conclusions plausible and coherent?  Have alternative explanations been explored and discounted?  Does this enhance understanding of the research topic?  Are the implications of the research clearly defined?  Is there adequate discussion of any limitations encountered?  *Adequate*  *Inadequate*  *Not sure* | This section is self-explanatory. | | | | | | |
| **Ethics** | | | | | | | |
| 14. How clear and coherent is the reporting of ethics?  For example:  Have ethical issues been taken into consideration?  Are they adequately discussed e.g. do they address consent and anonymity?  Have the consequences of the research been considered i.e. raising expectations, changing behaviour?  Was the study approved by an ethics committee?  *Appropriate*  *Inappropriate*  *Not sure/not reported* | All qualitative research has ethical considerations and these should be considered within any research report. Ideally there should be a full discussion of ethics, although this is rare because of space limitations in peer-reviewed journals. If there are particularly fraught ethical issues raised by a particularly sensitive piece of research, then these should be discussed in enough detail that the reader is convinced that every care was taken to protect research participants.  Any research with human participants should be approved by a research ethics committee and this should be reported. | | | | | | |
| **Overall assessment** | | | | | | | |
| As far as can be ascertained from the paper, how well was the study conducted? (see guidance notes)  *++*  *+*  *−* | Grade the study according to the list below:   - ++ All or most of the checklist criteria have been fulfilled; where they have not been fulfilled the conclusions are very unlikely to alter. - + Some of the checklist criteria have been fulfilled, where they have not been fulfilled, or not adequately described, the conclusions are unlikely to alter. - – Few or no checklist criteria have been fulfilled and the conclusions are likely or very likely to alter. | | | | | | |
