# Supplementary material for: Exploring the barriers and facilitators to the uptake of smoking cessation services for people in treatment or recovery from problematic drug or alcohol use: A qualitative systematic review
Source: PLoS One. 2023 Jul 13;18(7):e0288409. doi: 10.1371/journal.pone.0288409 (PMC10343091; doi:10.1371/journal.pone.0288409)
Supplement: S3 File — (DOCX) [file pone.0288409.s006.docx]

| Study identification: author, reference, year of  publication | **Berman *et al.* 2019** | **Bhuiyan *et al.* 2017** | **Fallin *et al.* 2016** | **Garner & Ratschen (2013)** | **Kathuria *et al.* 2019** | **McCool and Richter (2003** | **Pagano *et al.* 2016** | **Richter (2006)** | **Richter *et al.* 2002** | **Wilson *et al.* 2016** |
| --- | --- | --- | --- | --- | --- | --- | --- | --- | --- | --- |
| **Theoretical approach** | | | | | | | | | | |
| 1*. Is a qualitative approach appropriate?* | Appropriate | Appropriate | Appropriate | Appropriate | Appropriate | Appropriate | Appropriate | Appropriate | Appropriate | Appropriate |
| 2. *Is the study clear in what it seeks to do?* | Clear | Clear | Clear | Clear | Clear | Clear | Clear | Clear | Clear | Clear |
| 3. *How defensible/rigorous is the research design/methodology?* | Defensible | Defensible | Not sure | Not sure | Defensible | Not sure | Defensible | Not sure | Not sure | Defensible |
| 4. *How well was the data collection carried out?* | Appropriately | Appropriately | Inadequately reported | Inappropriately | Appropriately | Inappropriately | Inadequately reported | Not sure | Inadequately reported | Appropriately |

| Study identification: author, reference, year of publication | **Berman *et al.* 2019** | **Bhuiyan *et al.* 2017** | **Fallin *et al.* 2016** | **Garner & Ratschen (2013)** | **Kathuria *et al.* 2019** | **McCool and Richter (2003** | **Pagano *et al.* 2016** | **Richter (2006)** | **Richter *et al.* 2002** | **Wilson *et al.* 2016** |
| --- | --- | --- | --- | --- | --- | --- | --- | --- | --- | --- |
| **Trustworthiness** | | | | | | | | | | |
| 5*. Is the role of the researcher clearly described?* | Clearly described | Unclear | Clearly described | Clearly described | Clearly described | Clearly described | Unclear | Not described | Not described | Clearly described |
| 6. *Is the context clearly described?* | Clear | Clear | Clear | Clear | Clear | Clear | Clear | Unclear | Clear | Clear |
| **7***. Were the methods reliable?* | Reliable | Reliable | Reliable | Not sure | Reliable | Reliable | Reliable | Reliable | Reliable | Reliable |
| **Analysis** | | | | | | | | | | |
| 8. *Is the data analysis sufficiently rigorous?* | Not sure | Rigorous | Rigorous | Rigorous | Rigorous | Rigorous | Rigorous | Not sure | Rigorous | Rigorous |
| 9. *Is the data 'rich'?* | Rich | Rich | Poor | Poor | Rich | Rich | Not sure | Poor | Not sure | Rich |
| 10. *Is the analysis reliable?* | Reliable | Reliable | Reliable | Not sure | Reliable | Reliable | Reliable | Not reported | Reliable | Reliable |
| 11. *Are the findings convincing?* | Not convincing | Convincing | Not sure | Not convincing | Convincing | Convincing | Not convincing | Not convincing | Not convincing | Not sure |
| Study identification: author, reference, year of publication | **Berman *et al.* 2019** | **Bhuiyan *et al.* 2017** | **Fallin *et al.* 2016** | **Garner & Ratschen (2013)** | **Kathuria *et al.* 2019** | **McCool and Richter (2003** | **Pagano *et al.* 2016** | **Richter (2006)** | **Richter *et al.* 2002** | **Wilson *et al.* 2016** |
| 12. *Are the findings relevant to the aims of the study?* | Relevant | Relevant | Relevant | Partially relevant | Relevant | Relevant | Relevant | Relevant | Relevant | Relevant |
| 13. *Conclusions* | Inadequate | Adequate | Adequate | Inadequate | Adequate | Inadequate | Inadequate | Adequate | Adequate | Adequate |
| **Ethics** | | | | | | | | | | |
| 14. *How clear and coherent is the reporting of ethics?* | Appropriate | Appropriate | Not reported | Appropriate | Appropriate | Appropriate | Appropriate | Appropriate | Not reported | Appropriate |
| **Overall assessment** | | | | | | | | | | |
| *As far as can be ascertained from the paper, how well was the study conducted?* (see guidance notes) | + + | + + | + | - | + + | + + | + | **-** | + | + + |
