# Supplementary material for: Exploring the barriers and facilitators to the uptake of smoking cessation services for people in treatment or recovery from problematic drug or alcohol use: A qualitative systematic review
Source: PLoS One. 2023 Jul 13;18(7):e0288409. doi: 10.1371/journal.pone.0288409 (PMC10343091; doi:10.1371/journal.pone.0288409)
Supplement: Attachment — Submitted filename: Response to Reviewers.docx [file pone.0288409.s007.docx]

31st May 2023

Dear Johannes Stortz

PONE-D-23-04818 Exploring the barriers and facilitators to the uptake of smoking cessation services for people in treatment or recovery from problematic drug or alcohol use: A qualitative systematic review

We are delighted that our manuscript is potentially acceptable for publication in PLOS ONE, subject to minor revisions. Please find attached our revised manuscript and response to reviewer comment below. All the authors have read and approved the revised version of the manuscript which comprises original, unpublished material that has not been considered for publication elsewhere.

| Query |  | Response |
| --- | --- | --- |
| 1 | 1) The paper would greatly benefit from grammatical review. There is a dizzying number of commas and misplaced colons and semicolons that result in poor sentence structure throughout the paper. There are also a number of typos that are distracting (e.g., Page 9, Line 182, should refer to 420 database records rather than 420 databases, which would have been an extraordinary amount of work!) | We have carried out an extensive grammatical review of the paper. This included copy editing to reflect proper punctuations and improved sentence structure.  Also, we have changed the text on Page 9, Line 182 to 420 *database* records. |
| 2 | 2) The manuscript is quite long, even for a systematic review. I would suggest trimming the number of quotes presented in each section to only those that offer novel information in support of the theme. I would also encourage the authors to find ways to streamline the Discussion. | We have trimmed the number of quotes in the results section to reflect only those that offer novel information in support of the themes.  The discussion section has also been rephrased to reduce word count and the overall length of the manuscript. |
| 3 | 3) Page 2, line 21: This is the first place that the acronym PAD is introduced as referring to "problematic drug or alcohol use." This phrasing would suggest that the acronym should be PDA. Suggest rewording here and in other places where the acronym is defined. | Apologies for this oversight, this has been rectified. The acronym PAD has been replaced with *PDA* throughout the manuscript. |

Please do not hesitate to contact me if you wish to discuss anything further (contact details below signature).

We look forward to hearing from you in due course.

On behalf of all the authors,

Yours sincerely,

Eric Iyahen

ericiyahen@gmail.com  telephone: +234 (0) 634489544
