# Supplementary material for: Exploring the barriers and facilitators to the uptake of smoking cessation services for people in treatment or recovery from problematic drug or alcohol use: A qualitative systematic review
Source: PLoS One. 2023 Jul 13;18(7):e0288409. doi: 10.1371/journal.pone.0288409 (PMC10343091; doi:10.1371/journal.pone.0288409)
Supplement: Attachment — Submitted filename: Response to Reviewers.docx [file pone.0288409.s008.docx]

23rd May 2023

Dear Charlotte Lennox

PONE-D-23-04818R1 Exploring the barriers and facilitators to the uptake of smoking cessation services for people in treatment or recovery from problematic drug or alcohol use: A qualitative systematic review

We are delighted that our manuscript is potentially acceptable for publication in PLOS ONE, subject to minor revisions. Please find attached our revised manuscript and response to reviewer comment below. All the authors have read and approved the revised version of the manuscript which comprises original, unpublished material that has not been considered for publication elsewhere.

| Query |  | Response |
| --- | --- | --- |
| 1 | 1) Many thanks for attempting to address the reviewers' comments.  As academic editor I have reviewed the revisions and I think there is still more work to be undertaken in terms of reducing the overall length of the manuscript and shortening the length of sentences.  Please try and limit the use of quotes to one example per point, rather than in many places there are currently two quotes.  The discussion has not been sufficiently reduced and I would like this to be consolidated further. | We have carried out another round of copy editing to reflect proper punctuations and improved sentence structure. The length of sentences in the manuscript has been shortened because of this.  We have limited the use of quotes in the results section to only one quote to buttress each point made. Also, the discussion section has been further trimmed down to reduce word count and the overall length of the manuscript. |

Please do not hesitate to contact me if you wish to discuss anything further (contact details below signature).

We look forward to hearing from you in due course.

On behalf of all the authors,

Yours sincerely,

Eric Iyahen

ericiyahen@gmail.com  telephone: +234 (0) 634489544
